# The parity paradox: Differential effects on functional connectivity depending on APOEε4 genotype in middle age

**DOI:** 10.1002/alz70855_100368

**Published:** 2025-12-23

**Authors:** Bonnie H Lee, Andrew J McGovern, Stephanie E Lieblich, Selene V Mitchell, Jie Yin, Jonathan R Epp, Liisa AM Galea

**Affiliations:** ^1^ Centre for Addiction and Mental Health, Toronto, ON, Canada; ^2^ Centre for Addiction and Mental Health, Campbell Family Mental Health Research Institute, Toronto, ON, Canada; ^3^ University of British Columbia, Vancouver, BC, Canada; ^4^ University of Toronto, Toronto, ON, Canada; ^5^ University of Calgary, Calgary, AB, Canada

## Abstract

**Background:**

Females have a higher lifetime risk of Alzheimer's disease (AD) and show greater neuropathology and cognitive decline than males. Furthermore, possession of APOEε4 alleles confers greater risk and burden of AD in females than males. To better understand how AD affects females, female‐specific factors like parity (pregnancy and parenthood) are important to consider. Indeed, parity impacts brain aging in both humans and rodents, and has been associated with increased risk, greater neuropathology, and earlier age of onset for AD. We found that previous parity had opposite effects depending on genetic risk for late‐onset AD (in an humanized (h)APOEε4 rat model). Parity was associated with beneficial effects on brain health biomarkers in wildtype rats, yet detrimental effects in hAPOEε4 rats. This research explores the influences of parity and hAPOEε4 genotype on activation and connectivity of brain regions implicated in cognition and AD in middle‐aged rats.

**Method:**

Wildtype and hAPOEε4 rats were either nulliparous (never mothered) or primiparous (one‐time mothers). At middle age, rats were tested on a spatial working memory task then euthanized to examine functional connectivity using the immediate early gene, zif268, in response to memory retrieval. We examined activation of neurons across 19 brain regions to understand how previous parity and hAPOEε4 alter connectivity of neural networks.

**Results:**

Our findings demonstrate that previous primiparity in wildtype rats enhanced neural network efficiency and integration. In contrast, primiparity in hAPOEε4 rats was associated with increased fragmentation of the overall neural network. Primiparity in hAPOEε4 rats also reduced neural activation of key brain regions critical for memory, including the frontal cortex, dorsal striatum, nucleus accumbens, and retrosplenial cortex. Activation of subregions in the ventral hippocampus and granular retrosplenial cortex, both individually and in pairs, consistently predicted cognitive strategy use in a spatial working memory task, which depended on hAPOEε4 genotype.

**Conclusion:**

By revealing distinct patterns of network organization and regional importance, this study provides insight into the neural networks underlying cognition altered by primiparity and hAPOEε4 in middle‐aged female rats. These findings also demonstrate that considering within‐sex factors is crucial to unraveling the dynamic effects of AD risk on brain health outcomes.

